# Mapping network connectivity between internet addiction and residual depressive symptoms in patients with depression

**DOI:** 10.3389/fpsyt.2022.997593

**Published:** 2022-10-24

**Authors:** Hong Cai, Wei Bai, Yan Yue, Ling Zhang, Wen-Fang Mi, Yu-Chen Li, Huan-Zhong Liu, Xiangdong Du, Zhen-Tao Zeng, Chang-Mou Lu, Lan Zhang, Ke-Xin Feng, Yan-Hong Ding, Juan-Juan Yang, Todd Jackson, Teris Cheung, Feng-Rong An, Yu-Tao Xiang

**Affiliations:** ^1^Unit of Psychiatry, Department of Public Health and Medicinal Administration, Institute of Translational Medicine, Faculty of Health Sciences, University of Macau, Macao, Macao SAR, China; ^2^Centre for Cognitive and Brain Sciences, University of Macau, Macao, Macao SAR, China; ^3^Institute of Advanced Studies in Humanities and Social Sciences, University of Macau, Macao, Macao SAR, China; ^4^Guangji Hospital Affiliated to Soochow University, Suzhou, Jiangsu Province, China; ^5^Nanning Fifth People’s Hospital, Nanning, Guangxi, China; ^6^Department of Psychiatry, Lanzhou University Second Hospital, Lanzhou, Gansu, China; ^7^Department of Psychiatry, Xiamen Xianyue Hospital, Xiamen, Fujian Province, China; ^8^Department of Psychiatry, Chaohu Hospital, Anhui Medical University, Hefei, Anhui Province, China; ^9^School of Mental Health and Psychological Sciences, Anhui Medical University, Hefei, Anhui Province, China; ^10^School of Public Health, Lanzhou University, Lanzhou, Gansu Province, China; ^11^Department of Psychology, University of Macau, Macao, Macao SAR, China; ^12^School of Nursing, Hong Kong Polytechnic University, Kowloon, Hong Kong SAR, China; ^13^The National Clinical Research Center for Mental Disorders and Beijing Key Laboratory of Mental Disorders, Beijing Anding Hospital and the Advanced Innovation Center for Human Brain Protection, Capital Medical University, Beijing, China

**Keywords:** major depressive disorder, internet addiction, residential depressive symptoms, network analysis, central symptoms

## Abstract

**Background and aims:**

Depression often triggers addictive behaviors such as Internet addiction. In this network analysis study, we assessed the association between Internet addiction and residual depressive symptoms in patients suffering from clinically stable recurrent depressive disorder (depression hereafter).

**Materials and methods:**

In total, 1,267 depressed patients were included. Internet addiction and residual depressive symptoms were measured using the Internet Addiction Test (IAT) and the two-item Patient Health Questionnaire (PHQ-2), respectively. Central symptoms and bridge symptoms were identified via centrality indices. Network stability was examined using the case-dropping procedure.

**Results:**

The prevalence of IA within this sample was 27.2% (95% CI: 24.7–29.6%) based on the IAT cutoff of 50. IAT15 (“Preoccupation with the Internet”), IAT13 (“Snap or act annoyed if bothered without being online”) and IAT2 (“Neglect chores to spend more time online”) were the most central nodes in the network model. Additionally, bridge symptoms included the node PHQ1 (“Anhedonia”), followed by PHQ2 (“Sad mood”) and IAT3 (“Prefer the excitement online to the time with others”). There was no gender difference in the network structure.

**Conclusion:**

Both key central and bridge symptoms found in the network analysis could be potentially targeted in prevention and treatment for depressed patients with comorbid Internet addiction and residual depressive symptoms.

## Introduction

Recurrent depressive disorder (depression hereafter) is a major mental disorder (1, 2) that is associated with elevated risk of having comorbid addictive behaviors such as Internet addiction (IA) (3, 4). Persons with IA are defined as those who cannot control their Internet use and typically present several features including excessive Internet use, withdrawal symptoms, tolerance, and negative social repercussions (5).

Relations between addiction behaviors and depression appear to be bidirectional (6, 7). On one hand, depression may trigger addictive behaviors (8, 9). For example, preliminary evidence shows that addictive behaviors may alleviate certain depressive symptoms such as low mood (10), though, unfortunately depression contributes to an increased likelihood of problematic Internet use and IA (11–13). Conversely, excessive Internet use may also increase risk of depression (14). In addition, depression and IA share a common biological mechanism involving the 5HTTLRP gene (15, 16). Consistent with these lines of evidence, research on comorbidity has estimated the prevalence of comorbid IA in depressed patients is up to 58.6% (8, 9).

The COVID-19 pandemic and related challenges, such as social isolation and economic recession, are associated with increased risk of mental health problems (17) including depression and IA (18, 19). The pandemic has had a negative impact on health services in many countries including China. To elaborate, reduced medical service in tandem with strict public health measures (e.g., lockdowns) interfere with the capacity for clinically stable patients with psychiatric disorders including depression to attend regular check-ups and pursue routine physical exercise or social activity, hence increasing risk for psychiatric comorbidities including problematic Internet use and IA (20, 21).

In light of their frequent co-occurrence, it is essential to understand the association between IA and depression so that the risk of negative outcomes caused by IA in depressed patients including suicidal ideation (22); lowered well-being and poor academic performance (23, 24) can be reduced. However, to date, most associated studies have relied on analyses of relations between total scores on measures of depression and IA. Such approaches may overlook nuances in links between individual symptoms of these disorders and are counter to alternative perspectives that conceptualize psychiatric disorders as phenomena that arise as complex networks of mutually reinforcing symptoms (25, 26).

To address these concerns, network analysis offers a new statistical approach to conceptualizing and estimating interactions between various symptoms of psychiatric disorders (25). Network analysis can identify influential (central) symptoms within an entire symptom network (27, 28). Central symptoms may point to key mechanisms involved in triggering particular psychiatric disorders. Hence, treating these symptoms is associated with more effective outcomes (26). For example, a previous study on IA among Japanese adolescents with autism based on network analysis found that both concealment of Internet use and defensive and secretive behaviors were central symptoms (29). However, previous studies focused exclusively upon adolescents with IA or problematic smartphone use. Although network analyses can also examine comorbidities between psychiatric disorders/problems (30), to date, no published network studies have evaluated relations between IA and depressive symptoms among depressed patients.

To address this gap, the network structure of IA and residential depressive symptoms (RDS) was examined among clinically stabilized patients with depression.

## Materials and methods

### Participants

This study was part of a large-scale project on mental health status of clinically stable patients with depression during the COVID-19 pandemic conducted in outpatient departments of six tertiary psychiatric hospitals (31). Similar to other studies (32–35), the “Wenjuanxing” program embedded in the WeChat application was administered in this study. All outpatients treated in the participating hospitals were consecutively invited to complete this survey within the study period. Inclusion criteria were: (1) 18 years or older and (2) a diagnosis of recurrent depressive disorder based on the ICD-10 (36). Institutional Review Board (IRBs) of respective hospitals approved the study protocol, and all participants signed the online electronic informed consent.

### Measurements

A data collection form designed for this study was used to collect information on socio-demographic characteristics. The self-report Internet Addiction Test (IAT) (37, 38) was used to measure IA. The IAT consists of 20 items, each scored on a frequency scale from “1” (rarely) to “5” (always). The Chinese version of the IAT has been validated with good psychometric properties, including a Cronbach’s alpha of α = 0.90 (37). The two items-Patient Health Questionnaire (PHQ-2) (39, 40) was used to measure residual depressive symptoms (RDS); each item was scored for frequency of occurrence from “0” (not at all) to “3” (almost every day). Psychometric properties of the Chinese version of the PHQ-2 have been found to be satisfactory, including a Cronbach’s alpha of α = 0.76 (40).

### Analytical procedure

#### Network structure

R software (41) was used to examine comorbidity of IA and RDS network symptoms. We computed polychoric correlations among all IAT and PHQ-2 items to assess the network edges. We also estimated the Graphical Gaussian Model (GGM) using R package *“qgraph”* (42), with GGM as a pairwise Markov random field (PMRF) model used for interval or ordinal data. Edges in the GGM reflect partial correlation coefficients, with thicker edges representing stronger relations between nodes. We estimated centrality indices of nodes to determine which symptoms were more influential (central) in the network (43). To further assess accuracy of centrality indexes, the bootstrap method was used to examine the stability of central index strength based on correlation stability coefficients (CS-coefficient). We set the CS-coefficient cutoff at 0.25 for all indexes because evidence indicates CS-coefficients are typically below 0.25 when centralities do not differ from one another (42).

In addition, we estimated bridge symptoms with the bridge function of R package *“networktools”* (44, 45). Following a previous study (30), bridge strength is the widely used index to examine symptoms that may stop activation spread between different psychiatric disorders/syndromes.

#### The relationship between network model and genders

An earlier study have found gender had a significant role on both depression and IA (46). Similar to other network analyses (47, 48), gender differences of network characteristics were examined with the R “*NetworkComparisonTest”* package (Version 2.2.1) (49). Gender differences in network structure, global strength and each specific edge were also examined.

## Results

### Study sample

In total, 1,298 patients were screened; 1,267 (97.6%) fulfilled the inclusion criteria and were included for analyses. Of these, 367 (29.0%) were men, 509 (40.2%) were married, and 1,006 (79.4%) had senior high school education or higher. The mean IAT score was 40.85 [standard deviation (SD) = 16.21] and prevalence of IA within this sample 27.2% (95% CI: 24.7–29.6%) based on the IAT cutoff of 50 (Table 1).

**TABLE 1:** Sample characteristics (*n* = 1,267).

Variables	
	***N* (%)**
Male gender	367 (29.0)
Married	509 (40.2)
Senior high school and above	1,006 (79.4)
	Mean (SD)
Age(year)	32.2 (15.2)
IAT score	40.85 (16.21)

SD, standard deviation; IAT, internet addiction test.

### Network structure

**FIGURE 1:**
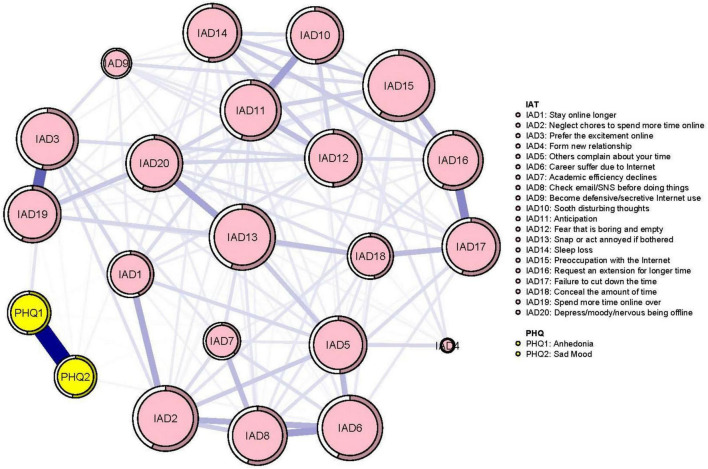
Network of residual depressive symptoms and internet addiction.

The mean predictability was 0.50 in the network model (Figure 1), which indicates that, on average, 50% of the variance in each node could be explained by nodes in the model. The network analysis indicated the connection between node PHQ1 (“Anhedonia”) and PHQ2 (“Sad mood”) was the strongest positive edge in the RDS community. In the IA community, the connection between IAT3 (“Prefer excitement online to the time with others”) and IAT19 (“Spend more time online over going out with others”) was the strongest positive edge, followed by edges between IAT16 (“Request an extension for longer time spent online”) and IAT17 (“Failure to cut down time spent online”) and between IAT6 (“Careers suffer due to Internet use”) and node IAT8 (“Check email/SNS before doing things”). In the RDS and IA network model, the connection between nodes PHQ1 (“Anhedonia”) and IAT3 (“Prefer excitement online to the time with others”) (average edge weight = 0.07) had the strongest positive edge, followed by connections between PHQ2 (“Sad mood”) and IAT6 (“Careers suffer due to Internet use”) (average edge weight = 0.02454), and between PHQ2 (“Sad mood”) and IAT14 (“Sleep loss due to late-night logins”) (average edge weight = 0.02345) (Figure 1 and Supplementary Tables 1, 2).

**FIGURE 2:**
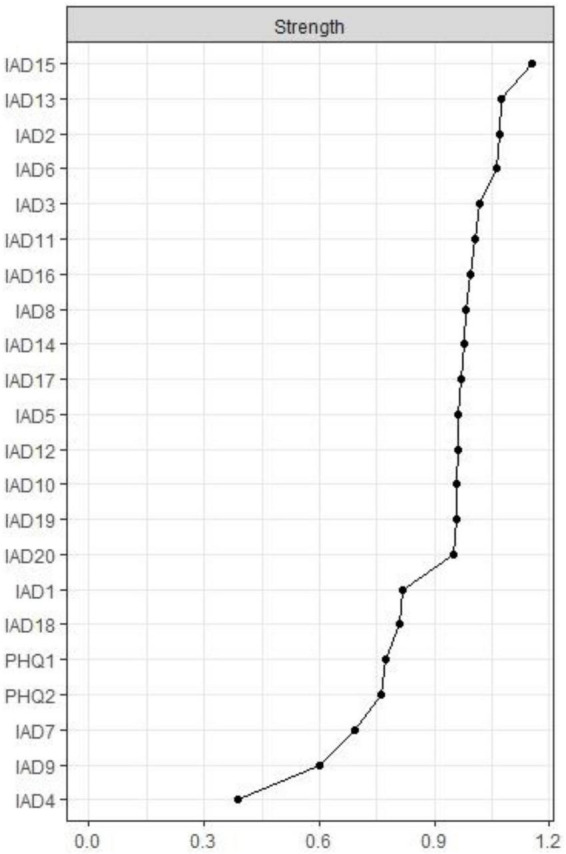
Centrality indices of network. PHQ-1: Anhedonia; PHQ-2: Sad Mood; IAT-1: Stay online longer; IAT-1: Stay online longer than you intend; IAT-2: Neglect chores to spend more time online; IAT-3: Prefer the excitement online to the time with others; IAT-4: Form new relationship with online users; IAT-5: Others complain about your time spend online; IAT-6: School grades suffer due to Internet use; IAT-7: Academic efficiency declines due to internet use; IAT-8: Check email/SNS before doing things you need to do; IAT-9: Become defensive/secretive Internet use; IAT-10: Sooth disturbing thoughts using the Internet; IAT-11: Anticipation for future online activities; IAT-12: Fear that life is boring and empty without the Internet; IAT-13: Snap or act annoyed if bothered without being online; IAT-14: Sleep loss due to late-night logins; IAT-15: Preoccupation with the Internet; IAT-16: Request an extension for longer time; IAT-17: Failure to cut down the time spend online; IAT-18: Conceal the amount of time spend online; IAT-19: Spend more time online over going out with others; IAT-20: Depress/moody/nervous being offline.

**FIGURE 3:**
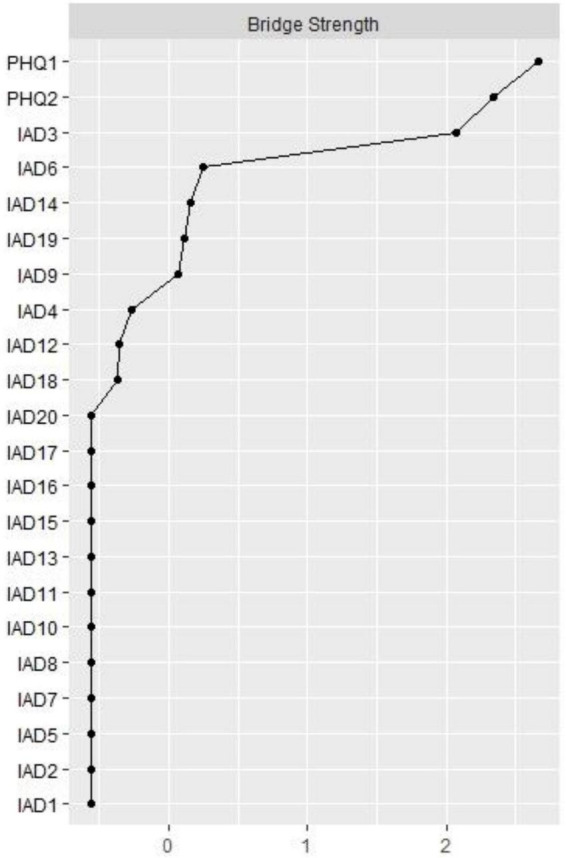
Bridge centrality indices of network. PHQ-1: Anhedonia; PHQ-2: Sad Mood; IAT-1: Stay online longer than you intend; IAT-2: Neglect chores to spend more time online; IAT-3: Prefer the excitement online to the time with others; IAT-4: Form new relationship with online users; IAT-5: Others complain about your time spend online; IAT-6: School grades suffer due to Internet use; IAT-7: Academic efficiency declines due to internet use; IAT-8: Check email/SNS before doing things you need to do; IAT-9: Become defensive/secretive Internet use; IAT-10: Sooth disturbing thoughts using the Internet; IAT-11: Anticipation for future online activities; IAT-12: Fear that life is boring and empty without the Internet; IAT-13: Snap or act annoyed if bothered without being online; IAT-14: Sleep loss due to late-night logins; IAT-15: Preoccupation with the Internet; IAT-16: Request an extension for longer time; IAT-17: Failure to cut down the time spend online; IAT-18: Conceal the amount of time spend online; IAT-19: Spend more time online over going out with others; IAT-20: Depress/moody/nervous being offline.

Node IAT15 (“Preoccupation with the Internet”) had the highest strength, followed by nodes IAT13 (“Snap or act annoyed if bothered without being online”) and IAT2 (“Neglect chores to spend more time online”) within the network (Figure 2), suggesting that these symptoms are most central for the association between RDS and IA in clinically stable depressed patients (Figure 2). In terms of bridge centrality, PHQ1 (“Anhedonia”) was the key bridge symptom linking RDS and IA, followed by PHQ2 (“Sad mood”) and IAT3 (“Prefer excitement online to the time with others”) (Figure 3).

**FIGURE 4:**
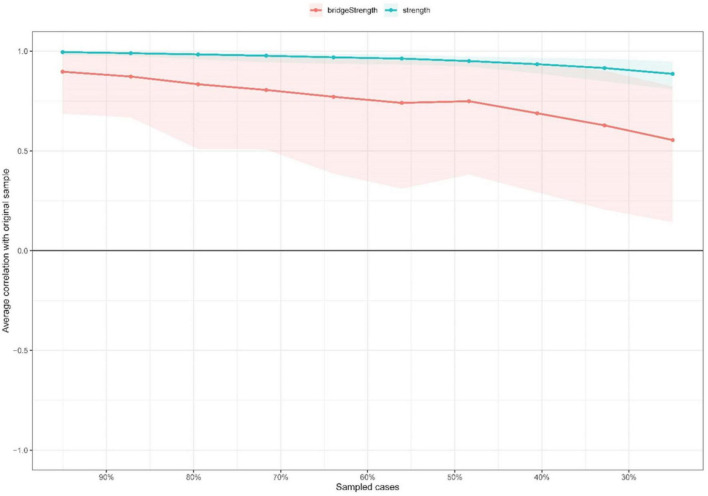
Stability of network structure by case dropping subset bootstrap. The x-axis represents the percentage of cases of the original sample used at each step. The y-axis represents the average of correlations between the centrality indices in the original network and the centrality indices from the re-estimated networks after excluding increasing percentages of cases. The lines indicate the correlations between strength and bridge strength.

Regarding network stability, centrality index strength had excellent stability with a CS-coefficient of 0.75, indicating that 75% of the sample could be dropped and the structure of the network would not change significantly (Figure 4). Supplementary Figure 1 shows that the bootstrapped 95% CIs for estimated edge weights were relatively narrow. The bootstrap difference test indicated most comparisons between edge weights were statistically significant (Supplementary Figure 1).

### The confounding effects of basic demographic data on internet addiction and residential depressive symptoms

Previous studies (50, 51) found that gender, age, marital status and education level had significant associations with IA in depressed patients. Following previous studies (52, 53), IA and RDS network models and structure indexes were re-calculated after controlling for age, marital status, gender, and education level. No significant structure change was found compared to the original network, after controlling for these factors (strength: r_s_ = 0.89 [0.80; 0.96]) (Supplementary Figure 2).

### Gender differences in the observed network model

No significant gender difference was identified in terms of network global strength (network strength: 9.84 for men; 10.15 for women; S = 0.31, *p* = 0.131), and edge weights (M = 0.16, *p* = 0.639; Supplementary Figures 3–5).

## Discussion

This was the first study to investigate relations of IA and RDS in a large sample of patients with depression from the perspective of network analysis. IAT15 (“Preoccupation with the Internet”), IAT13 (“Snap or act annoyed if bothered without being online”) and IAT2 (“Neglect chores to spend more time online”) were the most influential symptoms in the IA-RDS network model. Furthermore, bridge symptoms included PHQ1 (“Anhedonia”), PHQ2 (“Sad mood”) and IAT3 (“Prefer excitement online to the time with others”). Potential implications are discussed below.

Analyses identified node IAT15 (“Preoccupation with the Internet”) as the most influential symptom, which is consistent with proposed diagnostic criteria for IA in previous studies (54). Patients with IA often report initial experiences of increased preoccupation with the Internet; those with IA often think about Internet use when they are offline and fantasize about surfing the Internet even when they are attempting to concentrate on other tasks (55). Previous studies found that premorbid personality and cognitive dysfunctions are closely associated with increased risk for excessive Internet use and IA (56). Depressed patients with cognitive dysfunction may have difficulty exercising inhibitory control over Internet use (57, 58). Therefore, relevant treatments could target cognitive dysfunction as a means to improving impulse control and preoccupation with the Internet for depressed patients with comorbid IA.

Node IAT13 (“Snap or act annoyed if bothered without being online”) was also a central symptom within the IA-RDS network in clinically stable patients with depression. Depressed patients often become socially withdrawn and/or find interpersonal contacts to be overwhelming (59); hence for some of them, the Internet may provide an alternative, less burdensome means of experiencing social contact (60). However, it should be noted that IA and depression both have negative interpersonal effects, that could, in turn, perpetuate each of these problems (61). Another central symptom in the IA-RDS model was node IAT2 (“Neglect chores to spend more time online”), that aligns with commonly used IA criteria related to continued excessive use and loss of control of the Internet to the neglect of other responsibilities (62, 63). Our findings also converge with previous network analysis findings on problematic smartphone use in Chinese adolescents (64) and evidence that excessive screen time may trigger IA (65). In tandem with these data, one hypothesis that follows from this finding is that control processes are a critical influence on the prevention and treatment of IA in clinically stable patients with depression. It follows that efforts to reduce screen time and strengthen self-control may be useful targets in treating and preventing IA in patients with depression.

PHQ1 (“Anhedonia”), PHQ2 (“Sad mood”), and IAT3 (“Prefer the excitement online to the time with others”) were the key bridge nodes linking IA and RDS communities. These results dovetail with previous findings (66, 67) linking anhedonia and sad mood (i.e., diminished pleasure in normally enjoyable activities) to the etiology of IA. Specifically, anhedonia and sad mood are associated with lowered reward sensitivity and reduced ventral striatum responsivity to pleasant or beneficial stimuli (66–71). Furthermore, anhedonia has been found to predict increased risk for compulsive Internet use and addiction (72). Conversely, IA is related to lowered reward sensitivity, possibly due, in part, to reduced reward-related subcortical system activity (66, 73). In addition, misuse of the Internet, characterized by excessive time spent online, has been implicated as an influence on increased risk for depression (74–76).

Internet addiction-residual depressive symptoms network model results have potentially important implications for clinical practice in treating and preventing comorbid IA and depression. For instance, cognitive behavioral therapy (CBT) targeting central and/or bridge symptoms in this network through behavioral activation and cognitive restructuring may have utility in treating comorbidity in this population (57, 58). For patients with depression, psychosocial interventions targeting anhedonia may be helpful to prevent and treat comorbid IA (72). Alternately, a recent review concluded that most antidepressants have beneficial effects in reducing anhedonia (77). Strategies used to reduce symptoms related to sad mood and lack of pleasure and interest may have corresponding benefits in reducing IA symptoms when excessive internet use is a source for excitement.

Advantages of this study included use of network analysis, a multicenter study design and a large sample size. However, certain limitations need to be noted. First, causality between variables cannot be inferred because of the cross-sectional study design. Second, this study was conducted among clinically stable patients with depression, a focus that limits generalizability of findings to depressed patients in other stages (e.g., initial onsets). Third, the study did not assess all depressive symptoms or comorbidities that may have influenced the observed network model to some extent. To elaborate, although an impressive 50% of the variance in each node could be explained by neighboring nodes, substantial remaining variance in IA-RDS model was not accounted for by measured variables. Fourth, confounding effects of basic demographic data on IA and RDS were statistically controlled for in the network analysis. For logistical reasons, however, certain disease-related factors linked to psychiatric comorbidities, such as use of psychotropic medications, were not recorded. Consequently, unmeasured variables associated with IA and RDS such as other depressive symptoms (e.g., lack of energy, impaired concentration, inappropriate guilt), suicidality, poor sleep quality, severity of depression and other psychiatric comorbidities (e.g., anxiety disorders) (58, 78–80) should be considered in extensions of the current work.

In conclusion, this study identified preoccupation with the Internet, neglect chores to spend more time online, and requests for extensions to spend more time online as the most central nodes and Anhedonia, Sad mood, and Prefer excitement online to the time with others as key bridge nodes in an IA-RDS network model of clinically stable patients with depression. With the help of certain applications targeting Internet use [e.g., tools that help to block or limit amounts of time and access to the Internet, social media or gaming websites (81, 82)] and interventions to reduce emotional distress, clinically stable depressed patients could reduce their risk for IA (83). Additionally, family members and guardians should also help patients control use of internet activities and reduce the negative influence of unsafe and unhealthy information from the Internet (84, 85).

## Data availability statement

The Institutional Review Board (IRB) of participating hospitals that approved the study prohibits the authors from making the research data set publicly available. Readers and all interested researchers may contact Y-TX (xyutly@gmail.com).

## Ethics statement

The studies involving human participants were reviewed and approved by Beijing Anding Hospital. The patients/participants provided their written informed consent to participate in this study.

## Author contributions

F-RA and Y-TX: study design. HC, WB, XD, LiZ, LaZ, Y-CL, H-ZL, and F-RA: data collection, analysis, and interpretation. HC, TC, and Y-TX: drafting of the manuscript. TJ: critical revision of the manuscript. All authors approval of the final version for publication.
