# Supplementary material for: Mapping network connectivity between internet addiction and residual depressive symptoms in patients with depression
Source: Front Psychiatry. 2022 Oct 24;13:997593. doi: 10.3389/fpsyt.2022.997593 (PMC9638086; doi:10.3389/fpsyt.2022.997593)
Supplement: Supplementary file 1 [file Data_Sheet_1.docx]

**Supplementary Material**

Table S1. Means, standard deviations, skewness, and kurtosis.

Table S2. Correlation matrix of the PHQ-2 and IAT items.

Figure S1. Nonparametric bootstrapped difference test.

Figure S2. Visual representation of the network after controlling for age, gender, marital status, and education.

Figure S3. Estimated network model for the association between residual depressive symptoms and Internet addiction in females and males.

Figure S4. Comparison of network centrality indices between females and males.

Figure S5. Comparison of network properties between females and males.

Table S1. Means, standard deviations, skewness, and kurtosis.

| Item | M | SD | Skewness | kurtosis |
| --- | --- | --- | --- | --- |
| IAT-1 | 2.66 | 1.27 | 0.19 | -0.18 |
| IAT-2 | 2.17 | 1.17 | 0.70 | -0.49 |
| IAT-3 | 2.32 | 1.28 | 0.58 | -0.80 |
| IAT-4 | 1.73 | 0.98 | 1.34 | 1.15 |
| IAT-5 | 2.15 | 1.26 | 0.79 | -0.53 |
| IAT-6 | 1.93 | 1.13 | 0.99 | -0.04 |
| IAT-7 | 1.77 | 1.05 | 1.24 | 0.58 |
| IAT-8 | 1.73 | 1.02 | 1.28 | 0.76 |
| IAT-9 | 2.34 | 1.29 | 0.56 | -0.80 |
| IAT-10 | 2.39 | 1.30 | 0.44 | -1.04 |
| IAT-11 | 2.00 | 1.18 | 0.92 | -0.23 |
| IAT-12 | 2.38 | 1.29 | 0.52 | -0.87 |
| IAT-13 | 1.81 | 1.05 | 1.14 | 0.40 |
| IAT-14 | 2.16 | 1.21 | 0.73 | -0.55 |
| IAT-15 | 2.03 | 1.08 | 0.76 | -0.38 |
| IAT-16 | 2.16 | 1.17 | 0.68 | -0.58 |
| IAT-17 | 1.87 | 1.10 | 1.08 | 0.21 |
| IAT-18 | 1.62 | 0.98 | 1.50 | 1.36 |
| IAT-19 | 1.97 | 1.22 | 1.03 | -0.06 |
| IAT-20 | 1.67 | 1.03 | 1.48 | 1.29 |
| PHQ-1 | 2.61 | 1.02 | 0.05 | -1.18 |
| PHQ-2 | 2.48 | 1.00 | 0.19 | -1.04 |

Table S2. Correlation matrix of the PHQ-2 and IAT items.

|  | IAT1 | IAT2 | IAT3 | IAT4 | IAT5 | IAT6 | IAT7 | IAT8 | IAT9 | IAT10 | IAT11 | IAT12 | IAT13 | IAT14 | IAT15 | IAT16 | IAT17 | IAT18 | IAT19 | IAT20 | PHQ1 | PHQ2 |
| --- | --- | --- | --- | --- | --- | --- | --- | --- | --- | --- | --- | --- | --- | --- | --- | --- | --- | --- | --- | --- | --- | --- |
| IAT1 |  |  |  |  |  |  |  |  |  |  |  |  |  |  |  |  |  |  |  |  |  |  |
| IAT2 | 0.22 |  |  |  |  |  |  |  |  |  |  |  |  |  |  |  |  |  |  |  |  |  |
| IAT3 | 0.10 | 0.08 |  |  |  |  |  |  |  |  |  |  |  |  |  |  |  |  |  |  |  |  |
| IAT4 | 0.007 | 0 | 0 |  |  |  |  |  |  |  |  |  |  |  |  |  |  |  |  |  |  |  |
| IAT5 | 0.10 | 0.13 | 0.06 | 0.10 |  |  |  |  |  |  |  |  |  |  |  |  |  |  |  |  |  |  |
| IAT6 | 0.01 | 0.20 | 0 | 0 | 0.19 |  |  |  |  |  |  |  |  |  |  |  |  |  |  |  |  |  |
| IAT7 | 0.01 | 0.09 | 0.06 | 0.06 | 0 | 0.09 |  |  |  |  |  |  |  |  |  |  |  |  |  |  |  |  |
| IAT8 | 0.03 | 0.14 | 0 | 0 | 0.02 | 0.25 | 0.17 |  |  |  |  |  |  |  |  |  |  |  |  |  |  |  |
| IAT9 | 0.05 | 0 | 0.08 | 0 | 0.05 | 0.02 | 0.01 | 0.01 |  |  |  |  |  |  |  |  |  |  |  |  |  |  |
| IAT10 | 0.09 | 0.02 | 0 | 0.07 | 0.02 | 0.05 | 0.01 | 0.02 | 0.04 |  |  |  |  |  |  |  |  |  |  |  |  |  |
| IAT11 | 0 | 0.04 | 0 | 0.02 | 0 | 0 | 0.02 | 0.03 | 0.06 | 0.24 |  |  |  |  |  |  |  |  |  |  |  |  |
| IAT12 | 0.08 | 0 | 0.10 | 0 | 0 | 0 | 0 | 0 | 0.06 | 0.12 | 0.17 |  |  |  |  |  |  |  |  |  |  |  |
| IAT13 | 0 | 0.02 | 0 | 0.03 | 0.14 | 0.01 | 0.09 | 0.07 | 0 | 0 | 0.08 | 0.04 |  |  |  |  |  |  |  |  |  |  |
| IAT14 | 0.06 | 0.05 | 0.02 | 0.006 | 0.04 | 0.06 | 0 | 0.04 | 0.02 | 0.11 | 0 | 0.13 | 0.05 |  |  |  |  |  |  |  |  |  |
| IAT15 | 0 | 0 | 0 | 0.06 | 0.02 | 0.06 | 0 | 0.06 | 0.09 | 0.05 | 0.12 | 0.04 | 0.05 | 0.12 |  |  |  |  |  |  |  |  |
| IAT16 | 0.04 | 0.07 | 0.01 | 0 | 0.03 | 0 | 0 | 0 | 0.02 | 0.05 | 0 | 0.10 | 0 | 0.13 | 0.19 |  |  |  |  |  |  |  |
| IAT17 | 0 | 0 | 0 | 0 | 0.02 | 0.06 | 0 | 0.09 | 0.06 | 0 | 0.06 | 0.001 | 0 | 0.03 | 0.08 | 0.31 |  |  |  |  |  |  |
| IAT18 | 0 | 0 | 0 | 0 | 0.07 | 0.03 | 0.02 | 0.06 | 0 | 0 | 0.04 | 0 | 0.16 | 0 | 0.05 | 0 | 0.17 |  |  |  |  |  |
| IAT19 | 0 | 0 | 0.42 | 0 | 0.002 | 0 | 0 | 0 | 0 | 0.05 | 0 | 0.01 | 0.10 | 0.03 | 0.06 | 0.03 | 0 | 0.08 |  |  |  |  |
| IAT20 | 0 | 0 | 0 | 0.02 | 0 | 0.01 | 0.04 | 0.01 | 0 | 0 | 0.11 | 0.11 | 0.23 | 0.03 | 0.07 | 0.01 | 0.07 | 0.08 | 0.14 |  |  |  |
| PHQ1 | 0 | 0 | 0.07 | -0.01 | 0 | 0.002 | 0 | 0 | 0 | 0 | 0 | 0.01 | 0 | 0 | 0 | 0 | 0 | 0 | 0.02 | 0 |  |  |
| PHQ2 | 0 | 0 | 0.02 | 0 | 0 | 0.02 | 0 | 0 | 0.02 | 0 | 0 | 0 | 0 | 0.02 | 0 | 0 | 0 | -0.006 | 0.01 | 0 | 0.67 |  |

Figure S1. Nonparametric bootstrapped difference test.

A:


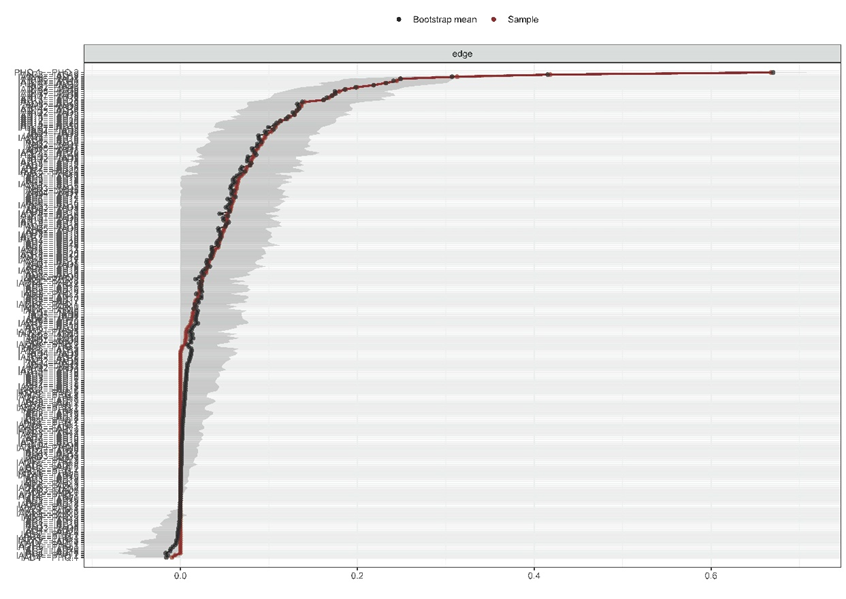


B:


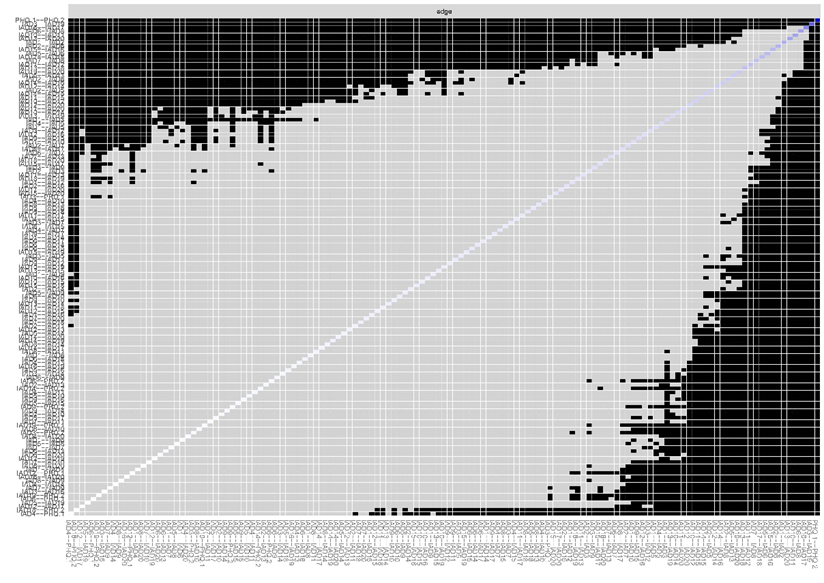


Figure S1. Panel A and Panel B: Nonparametric bootstrapped difference test for edge. Panel A: For this, 95% bootstrapped confidence interval (CI) for edge-weights are constructed based on the normal variance in the bootstrapped sample. Based on the range of these CIs indicated that a wide interval represents low stability, and a narrow interval represents high stability. Panel B: Bootstrapped difference tests between edge weights in the network. Gray boxes indicate edges that do not significantly differ from one-another. Black boxes represent edges with significant difference from one another. Blue boxes in the edge-weight plot indicate positive correlations.

Figure S2. Visual representation of the network after controlling for age, gender, marital status, and education.


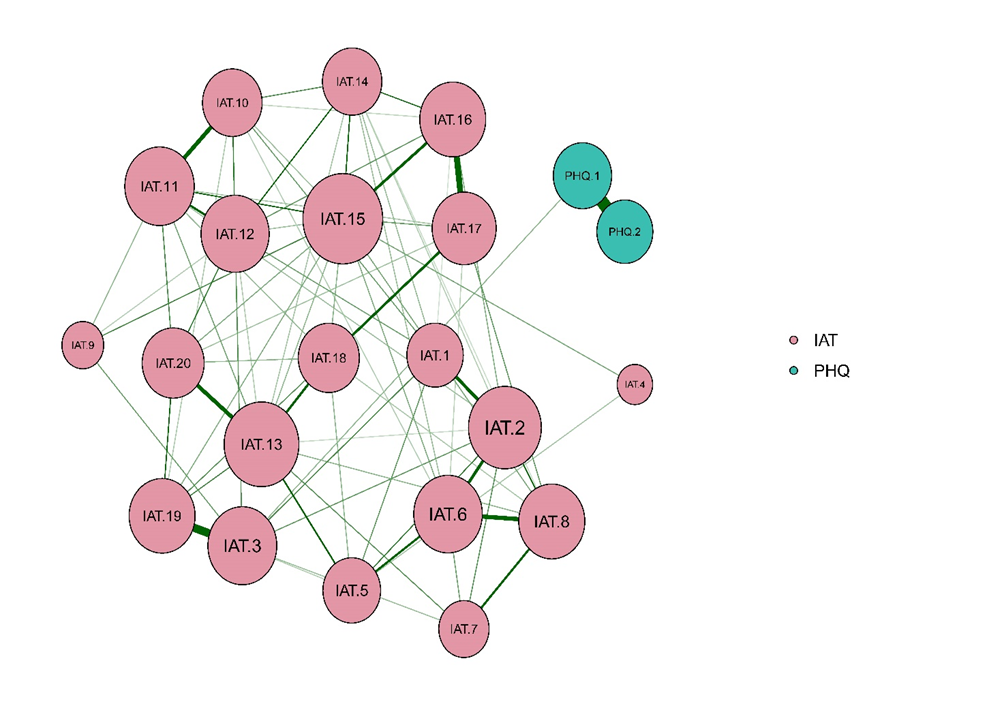


Figure S3. Estimated network model for the association between residual depressive symptoms and Internet addiction in females and males.


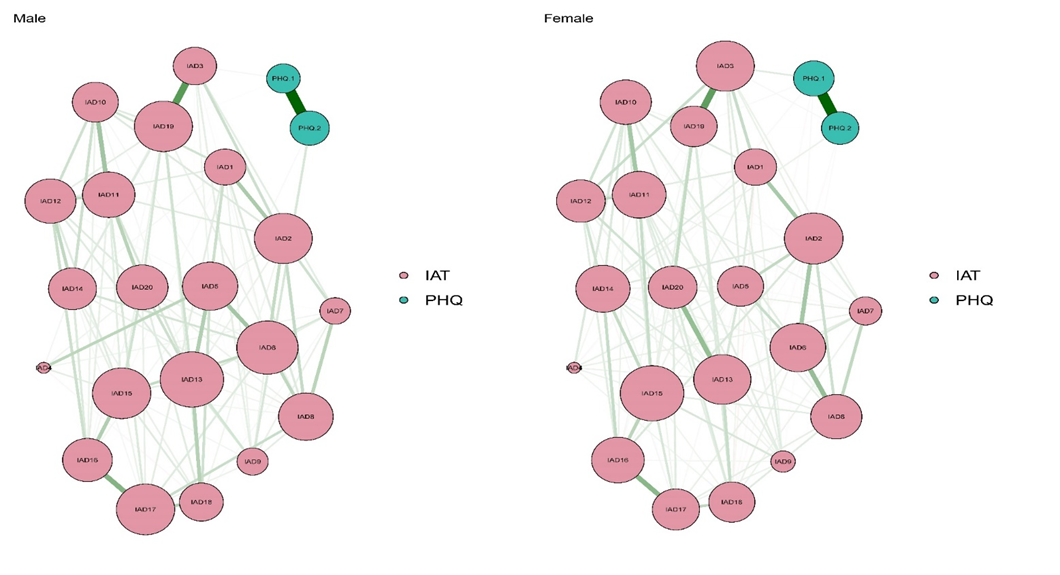


Figure S4. Comparison of network centrality indices between females and males.


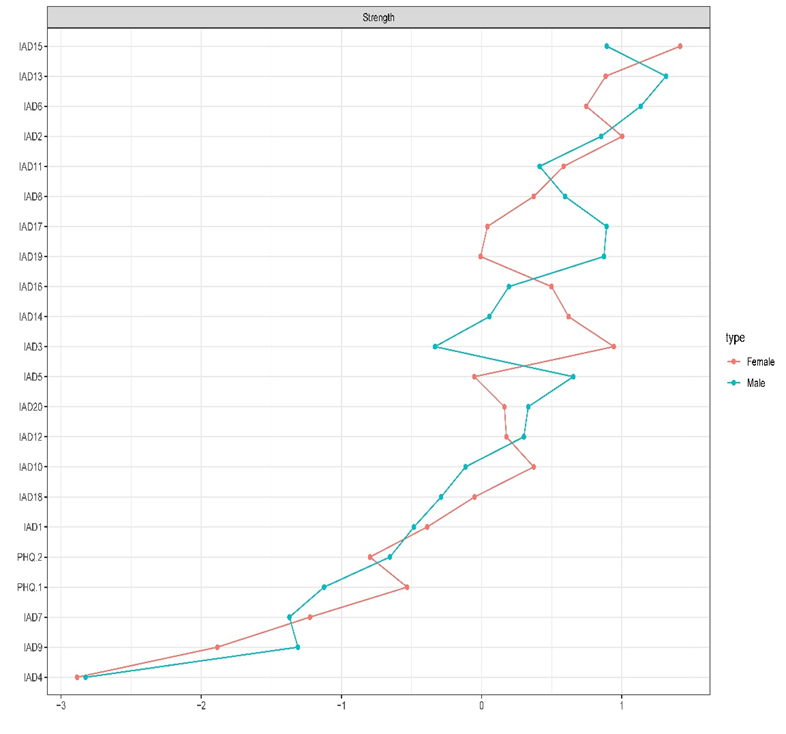


Figure S5. Comparison of network properties between females and males.


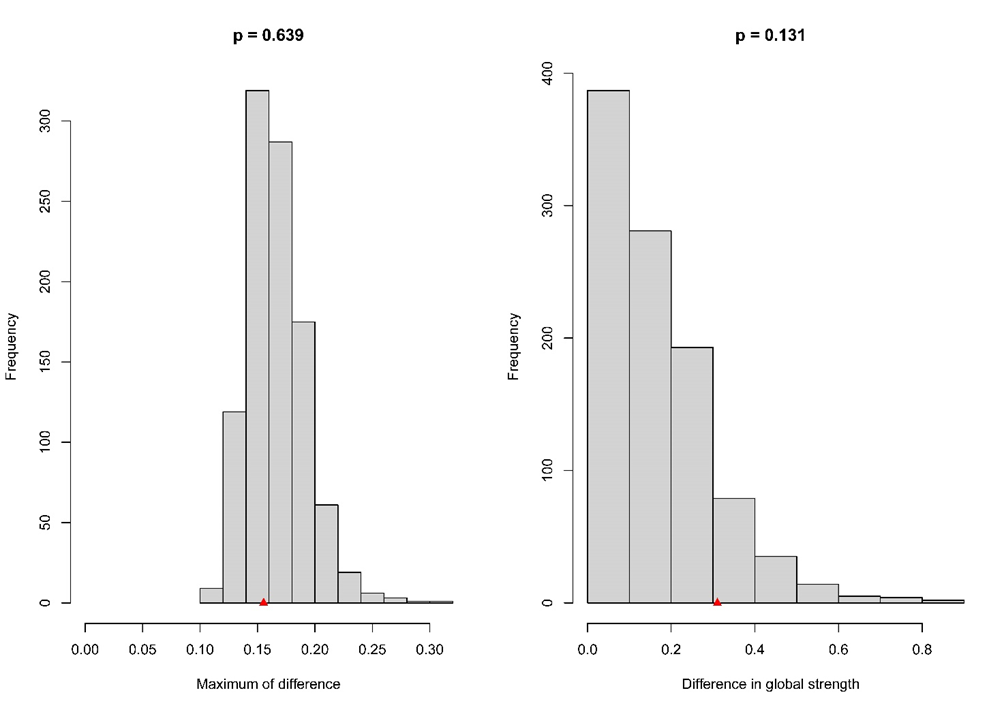


Node: Left Panel: A plot of bootstrap value of the maximum difference in any of the edge weights (1000 permutations). The difference was not significant (M=0.16, p=0.639).

Right Panel: A plot of bootstrap value of the difference in network global strength. The difference was not significant (network strength among male participants: 9.84; among female participants: 10.15; S: 0.31, p=0.131). Invariance in edge weights was tested using the permutation test, generating sets of p values for each edge-edge comparison. Holm-Bonferroni corrected p values were all >0.05 indicating absence of significant differences.
